# Programmable optical meta-holograms

**DOI:** 10.1515/nanoph-2023-0544

**Published:** 2023-10-02

**Authors:** Jing Cheng Zhang, Yubin Fan, Jin Yao, Mu Ku Chen, Shirong Lin, Yao Liang, Borui Leng, Din Ping Tsai

**Affiliations:** Department of Electrical Engineering, City University of Hong Kong, Kowloon, Hong Kong SAR, China; State Key Laboratory of Terahertz and Millimeter Waves, City University of Hong Kong, Kowloon, Hong Kong SAR, China; Centre for Biosystems, Neuroscience, and Nanotechnology, City University of Hong Kong, Kowloon, Hong Kong SAR, China

**Keywords:** metasurface, meta-device, meta-hologram, programmable optical meta-hologram

## Abstract

The metaverse has captured significant attention as it provides a virtual realm that cannot be experienced in the physical world. Programmable optical holograms, integral components of the metaverse, allow users to access diverse information without needing external equipment. Meta-devices composed of artificially customized nano-antennas are excellent candidates for programmable optical holograms due to their compact footprint and flexible electromagnetic manipulation. Programmable optical meta-holograms can dynamically alter reconstructed images in real-time by directly modulating the optical properties of the metasurface or by modifying the incident light. Information can be encoded across multiple channels and freely selected through switchable functionality. These advantages will broaden the range of virtual scenarios in the metaverse, facilitating further development and practical applications. This review concentrates on recent advancements in the fundamentals and applications of programmable optical meta-holograms. We aim to provide readers with general knowledge and potential inspiration for applying programmable optical meta-holograms, both intrinsic and external ways, into the metaverse for better performance. An outlook and perspective on the challenges and prospects in these rapidly growing research areas are provided.

## 1 Introduction

**Figure 1:**
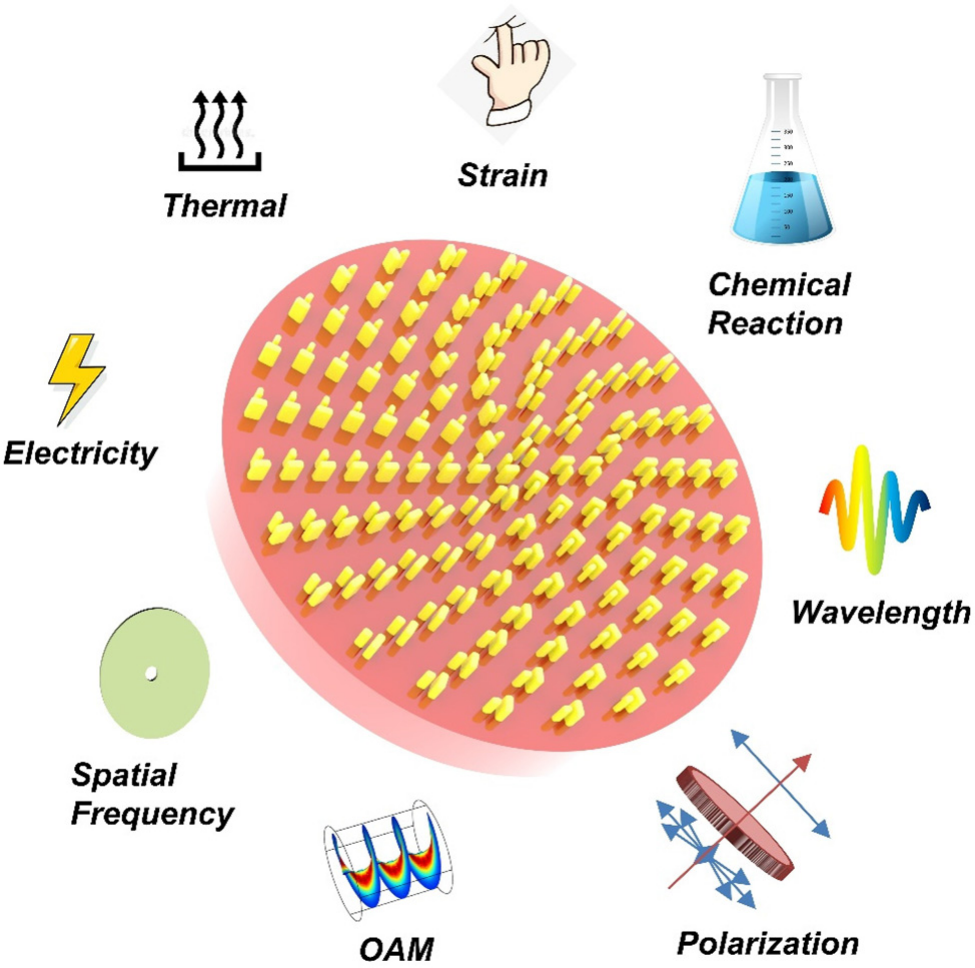
Versatile programmable meta-holograms are promoting the development of the metaverse.

The metaverse is a digitally created virtual environment that can interact with and outperform the real world. Media, such as photos and videos, are represented as holograms in the metaverse. The virtual environment is accessible via smartphones and virtual or augmented reality devices. Commercially available devices are very bulky and limit metaverse development. Metasurface provides an excellent solution to solve the problem of bulky and oversize. It is much easier to assemble metaverse systems or equipment due to the advantages of being more flat, ultrathin, and compact than conventional optical components. Composed of sub-wavelength artificial meta-antennas, the metasurface platform could accurately manipulate the wavefront of electromagnetic waves in sub-wavelength resolution [1–6]. High image quality and miniaturized size of devices can thus be achieved, promoting the development of display and data storage for the metaverse [7–12]. Meta-holograms are fabricated by the complementary metal–oxide semiconductor (CMOS) process. The fabrication process fixes the functions of meta-holograms and limits their practical applications. Programmable meta-holograms have recently received considerable attention, showing their potential to achieve ultra-high storage capacity and tunable features for holographic information. This can be attributed to the rapid development of tunable meta-devices in recent years [13–20]. Programmable meta-holograms can restore and reconstruct different holographic images by imposing external excitations, such as electricity and thermal, and altering the input/output wave by polarization and orbital angular momentum (OAM). Examples of different tuning methods associated with the programmable meta-hologram are illustrated in Figure 1.

Several reviews have been published on meta-holograms [21–24]. Less attention is focused on the recent development of programmable meta-holograms. This review will show the following parts. We present a brief introduction to the principles of the meta-hologram, followed by their versatile applications. The recent progress of the programmable meta-hologram is introduced in detail. We conclude the review with a discussion of the present challenges in this field and offer our perspective on future work, especially for the metaverse.

## 2 Principles of meta-holograms

**Figure 2:**
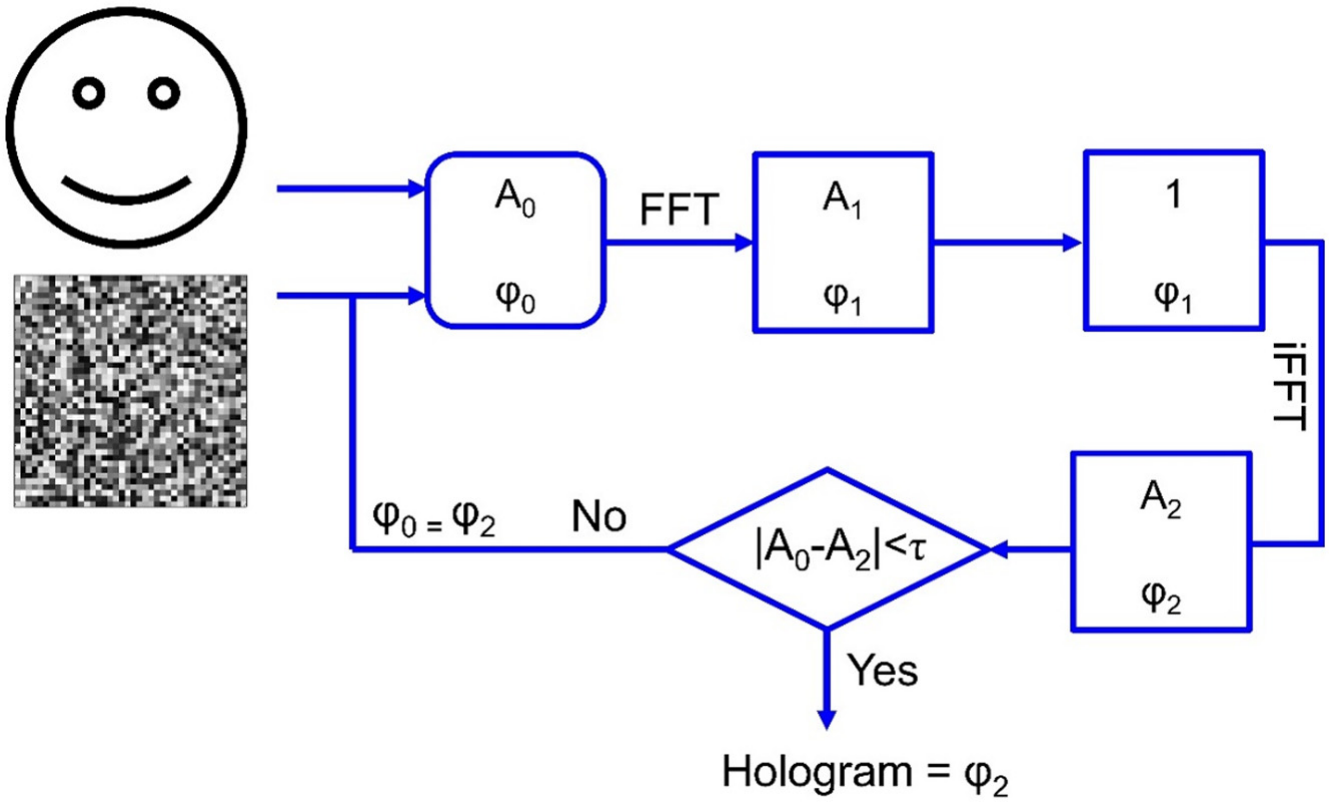
Flow chart of the GS algorithm.

The phase information reconstructing the meta-hologram could be calculated by a variety of techniques, including the Gerchberg–Saxton (GS) algorithm [25, 26], Yang-Gu algorithm [27], and Fresnel transform algorithms [28]. The GS algorithm is widely regarded as the most favored approach for generating phase-only holograms. It leverages the power of Fourier transformations to extract the critical phase information from a given set of images, making it a highly effective solution. By repeatedly utilizing the fast Fourier transformation (FFT) and inverse fast Fourier transformation (IFFT), as well as imposing the necessary constraints (*τ*) on the hologram and holographic image planes, the final phase distribution can be obtained. To be precise, there are usually five procedures shown in Figure 2. The algorithm’s loop will first begin the initial computation with the target image’s amplitude (*A*_0_) and a random phase distribution (*φ*_0_). Second, based on the idea that fields propagate from the holographic picture plane to the object plane, we compute the FFT of the original field. Only the phase (*φ*_1_) is kept, and the amplitude (*A*_1_) is set as uniform. Third, to determine the amplitude (*A*_2_) and phase (*φ*_2_) of the reconstructed holographic picture, we will compute the IFFT of the optical field based on how the field propagates from the object to the holographic image planes. Fourth, we will evaluate whether to proceed with another iteration by comparing the difference between *A*_2_ and *A*_0_ with predefined limits. If the difference exceeds these limits, the phase of the reconstructed holographic image (*φ*_2_) will be combined with the amplitude *A*_0_. The loop will be repeated until the difference falls within the specified bounds. After several loops, the phase-only hologram can be created.

Besides phase, light has many degrees of freedom, such as amplitude, wavelength, and polarization, which will inevitably be lost during the creation of phase-only holograms. It is an essential research topic to develop the ability to take full use of the capability of light. Metasurface, composed of subwavelength resonators, offers an excellent platform to overcome the limitation of information loss by enabling the modulation of amplitude, phase, polarization, and other high-dimensional optical light characteristics, thereby opening up a virtually limitless range of possibilities for hologram creation [29–34]. In recent years, numerous deep learning techniques [35, 36] have been explored for developing meta-holograms to achieve faster and superior performance compared to GS algorithms. For example, Li. et al. demonstrated a novel 3-D deep learning inversion methodology [37] to solve electromagnetic inverse scattering (EMIS) problems, characterized by its low computational cost, versatility, controllability, and scalability.

**Figure 3:**
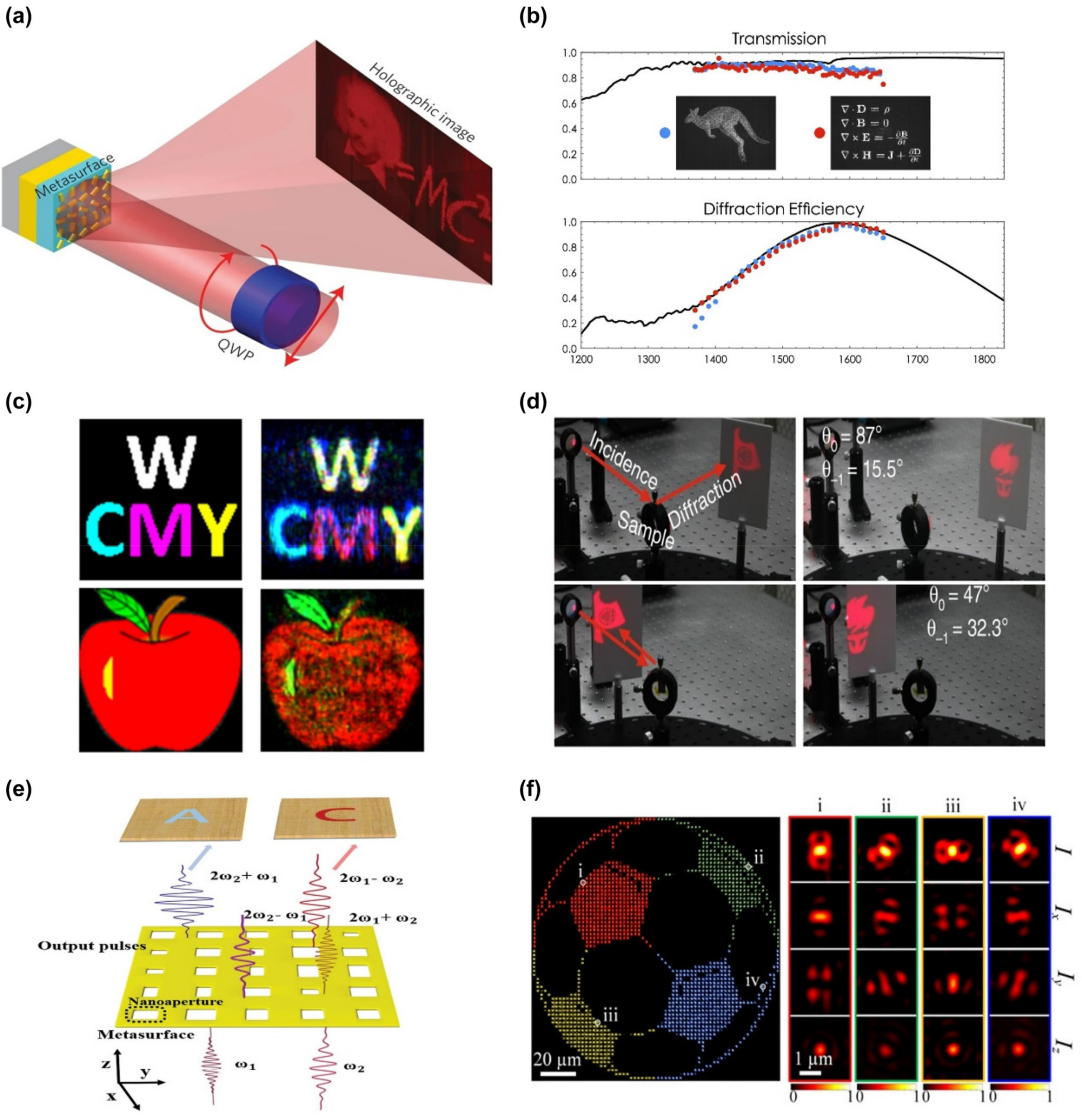
Multiple advantages of meta-holograms compared to traditional ones. (a) Schematic of the meta-hologram based on MIM metasurface. (b) The holographic image produced by the Huygens’ metasurface. The insert is the metasurface’s scanning electron microscope (SEM) image. (c) The full-color image reconstructed by the plasmonic metasurface. (d) Photographs of reconstructed ‘boat’ (blue) and ‘torch’ (red) images at different incident angles. (e) Schematic illustration of the nonlinear holography. (f) Principle of the 3D vectorial holography. (a) Reproduced with permission [44]. Copyright 2015, Nature Publishing Group. (b) Reproduced with permission [45]. Copyright 2016, Optica Publishing Group. (c) Reproduced with permission [46]. Copyright 2016, American Chemical Society. (d) Reproduced with permission [47]. Copyright 2018, Nature Publishing Group. (e) Reproduced with permission [49]. Copyright 2019, Wiley-VCH. (f) Reproduced with permission [54]. Copyright 2020, American Association for the Advancement of Science.

Metasurface-based holograms, also called meta-holograms, are expected to perform much better than traditional holograms, such as the high efficiency in a highly interactable way. The metasurface platform helps to consider the characteristics of lightness and high efficiency simultaneously [38–43]. The (metal–insulator–metal) MIM metasurface and Huygens’ metasurface can considerably improve the meta-hologram’s effectiveness. A meta-hologram with a MIM Pancharatnam–Berry (PB) phase metasurface that achieved a high efficiency of 80 % was demonstrated by Zhang et al. (Figure 3a) [44]. By altering the orientation angle of the meta-atoms in the same dimension, the metasurface based on the PB phase concept can provide two-phase control of a circular polarization beam. The polarization conversion efficiency can be about 90 % by fixing the phase difference between the reflected beams along the meta-atom axes. The reflection efficiency for both linear polarization bases can reach 80 %. A different plan based on generalized Huygens’ law was put up by Wang et al. to produce a high-efficiency meta-hologram (Figure 3b) [45]. Based on the theory providing a straightforward procedure to suppress the backward scattering, they discovered a diffraction efficiency of over 99 % and transmission efficiency of over 90 %.

In addition to their high efficiency, researchers also broaden the research to perform many other kinds of holograms which most traditional devices cannot accomplish, including full color, angle tolerance, etc. To recreate full-color images, Yang et al. developed a plasmonic meta-hologram (Figure 3c) [46]. By varying the amplitude and phase, holographic pictures can be produced with high clarity and minimal noise. A solution to the angle tolerance and bandwidth issues in meta-holograms was put out by Li et al. (Figure 3d) [47]. The unique optical diffraction meta-grating mechanism, inherently independent of the incidence angles and wavelengths, is used to achieve angle tolerance and gain high stability. The full-color hologram, the information density contained in the metasurface, and the hologram built using an optical diffraction meta-grating offer higher performance and stability. Meta-hologram can be expanded to nonlinear and 3D displays [48–53]. A nonlinear meta-hologram was conceptually proposed by Wang et al. (Figure 3e) [49]. Instead of using the incidence wavelengths, the holographic pictures are recreated using two harmonic wavelengths. Artificial intelligence may be a great technique to optimize the design when design complexity rises conveniently. Using an inverse method, Gu et al. created a 3D vectorial holography (Figure 3f) [54]. The phase hologram and vector field distribution help to recreate the intricate 3D holographic image with the aid of artificial neural networks.

Due to the advantages and the distinct features the traditional hologram cannot perform, meta-holograms present an up-and-coming solution for information restoration. Yet, their storage capacity and tunability are limited. Much attention has been directed toward developing programmable meta-holograms to enhance their capabilities. These can be broadly divided into intrinsically and externally programmable meta-holograms. The former involves altering the optical properties of the metasurface, while the latter focuses on modulating the incident light.

## 3 Intrinsically programmable meta-holograms

Through diverse external stimuli, programmable meta-holograms can be realized in real-time using metasurfaces made of reconfigurable meta-atoms [55]. The use of meta-holograms in adjustable visual display, high-capacity information storage, and encryption will considerably increase with the development of such a dynamic meta-hologram platform. In this section, we will introduce intrinsically programmable meta-holograms. The tuning methods include electricity, thermal, strain, chemical reactions, etc.

### 3.1 Electricity

**Figure 4:**
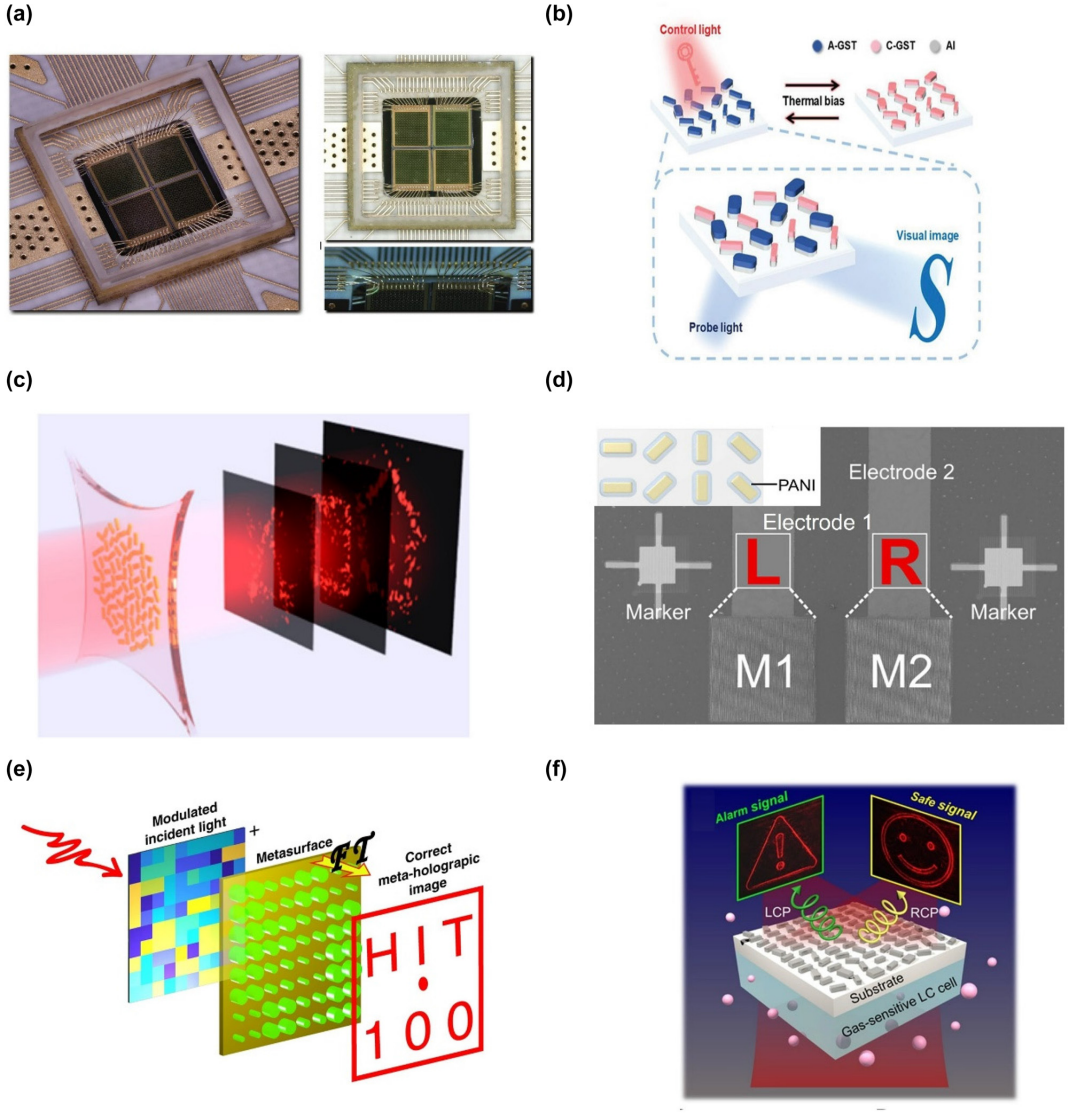
Examples of intrinsically programmable meta-hologram. (a) Upper panel: working principles of the programmable metasurface. Lower panel: the perspective and top view of the metasurface chip. (b) Working scheme (left panel) and design principle (right panel) of the hybrid state meta-hologram. (c) Overview of the stretchable metasurface hologram device. (d) Working principle of the electrochemically controlled metasurface. (e) Illustration of the programmable meta-hologram based on SLM. (f) Schematic of the gas sensor based on meta-hologram. (a) Reproduced with permission [61]. Copyright 2020, Nature Publishing Group. (b) Reproduced with permission [68]. Copyright 2021, Wiley-VCH. (c) Reproduced with permission [84]. Copyright 2017, American Chemical Society. (d) Reproduced with permission [94]. Copyright 2021, American Association for the Advancement of Science. (e) Reproduced with permission [95]. Copyright 2020, Nature Publishing Group. (f) Reproduced with permission [100]. Copyright 2021, American Association for the Advancement of Science.

There are advancements in programmable applications if the external electric stimulus could tune the metasurface, for instance, electronics [56], communication [57, 58], robots [59], etc. Holographic images can be made indefinitely flexible and compactly using the electrically adjustable approach. The metasurface’s unit cells each contain an electric diode switch. Their optical responses can be independently controlled by altering the biased voltages across these active components using a field programmable gate array (FPGA). One example is the 1-bit coding metasurface-based programmable hologram that Zhang et al. demonstrated [60]. Each meta-atom phase could be changed from 0 to π by electrically tuning the diodes built into the metasurface using an FPGA. Unlimited phase-only holographic images could be displayed in real-time by sensibly modulating the digital unit cells. It is the first phase-modulation programmable meta-hologram at microwave frequencies and opens the door for expanding programmable meta-holograms to include numerous levels of intricate amplitude modulations. Venkatesh’s team later developed a programmable terahertz holographic metasurface built on CMOS chips (Figure 4a) [61]. Five hundred seventy-six unit cells comprise the chip’s 2 × 2 metasurface array, individually addressable and digitally programmable using an 8-level control. Because FPGA addresses each switch individually, a C-shaped split-ring structure with various orientations and gaps can be created by integrating eight complimentary metal-oxide-semiconductor switches in a ring structure. The orientations and gaps can independently adjust the transmitted phase and amplitude. As a result, real-time switching of the 8-level complex amplitude holographic images is possible.

Employing liquid crystals (LCs) is an appealing option for an electrically programmable meta-hologram in the optical domain. LCs have a reliable, quick, and reversible response to external electric fields and minimal optical loss [62]. An LCs-supported dynamic PB phase-based meta-hologram was proposed by Rho et al. [63]. Through various combinations of phase and amplitude variances between left and right circular polarizations, nine holographic images with multiple polarization states could be switched. Depending on the biased voltage, the LCs layer at the rear of the metasurface acts as a dynamic polarization analyzer to block one or more holographic pictures. Involving the LC modulator instead of a combination of polarizer and retarder enables reconfigurability and programmability of the electrically tunable optical security platform while simplifying the combination and adjustment of optical components. They also showed off a meta-hologram that can be programmed and have five switchable holographic graphics. Five voltage levels applied on the LCs are used to choose one of the images. It is a solid security platform since it uses biased voltages to encrypt the numbers. A novel picture display method identified by a metasurface array was developed by Liu et al. [64]. Each metasurface is spaced with LCs in even or odd columns, and the others are spaced with polymethyl methacrylate. As the biased voltages vary, the refractive index of LCs changes, resulting in a variable phase difference between neighboring columns of the metasurface. When the phase difference results from constructive interference between the surrounding columns, the intensity of the reflection will be at its highest. When there is destructive interference, it will vanish. FPGA allows the biased voltages loaded on each metasurface to be dynamically changed. The benefits include excellent reversibility and high tuning speed on the millisecond time scale.

Every metasurface functioning as a unit cell rather than a subwavelength nano-antenna can significantly improve metasurface adjustability. Imaging precision will be further enhanced by the ability of FPGA to independently control each nano-antenna constructed of LCs of subwavelength scale as integration technology advances.

### 3.2 Thermal

Phase change materials like Ge_2_Sb_2_Te_5_ (GST) and vanadium dioxide (VO_2_) make suitable substrates for dynamic meta-holograms due to their notable difference in refractive index between their amorphous and crystalline phases or before and after insulator-to-metal transition [65–70]. A programmable meta-hologram made of phase-change materials can often switch between two holographic images. The variable phase change and adaptable tuning capabilities make it a desirable platform for programmable meta-holograms. For instance, Lee et al. used GST to show PB phase-based active meta-hologram with a potential tunable phase change of up to 10^15^ cycles [67]. A single unit cell contains two groups of nano-antennas with varied sizes, each in charge of reconstructing a particular holographic image. In the crystalline phase, the larger nano-antenna transmits cross-polarized light more efficiently than the smaller nano-antenna. The scenario is the exact reverse for the amorphous phase.

The same group quickly increased the switch performance of GST-based metasurface to three levels (Figure 4b) [68]. The co-existence of amorphous and crystalline GST-based nano-antennas introduces the third state. Unit cells consisting of four nano-antennas come in two different flavors. All four nano-antennas have the same structural size for one form of the unit cell. However, the other two types of nano-antennas do not. These two types are intended to behave differently in amorphous and crystalline states, and the nano-antenna also behaves in another way in a hybrid form, making it possible to build a three-level hologram based on the GST metasurface.

Another substance frequently used to create thermally biased programmable meta-holograms is VO_2_. As a phase transition metal oxide, the insulator-to-metal phase transformation could be quickly accomplished at a relatively low temperature (of about 67 °C). Additionally, it is appropriate for a broad spectrum, including terahertz and infrared wavelengths [71–73]. Zhang et al. showed a thermally dependent dynamic meta-holograph helped by VO_2_ [69]. Two sets of nano-antennas are present. The first group consists of gold-based metallic C-shaped split-ring resonators (CSRRs), while the second group comprises VO_2_-integrated CSRRs (V-CSRRs), which occupy the space left by the CSRRs. As VO_2_ heated up, it gradually became an insulator to a metal phase, shorting out the CSRRs and dynamically rebuilding various holographic images. The two sets will produce the letter “*H*” at lower temperatures in their pattern. The remaining CSRRs will have the letter “*H*” at high temperatures when VO_2_ in the metallic phase shorts out the CSRRs.

Thus, the thermal tuning method can work broadly from visible to THz, making switchable metasurfaces anticipated to facilitate cutting-edge research and intelligent applications about various tunable functionalities.

### 3.3 Strain

When stretched by external pressure, the stretchy substrate will impact the metasurface’s optical response. Most of the research was initially devoted to adjusting the optical sensitivity of the same nano-antennas throughout the entire metasurface and did not perform complex functions [74–78]. The stretchy substrate technique was then used to realize optical wavefront adjustment [79–83]. For instance, Agarwal et al. produced a stretchy substrate-based tunable meta-lens in the visible spectrum [83]. When extended by a factor of *s*, the focal length will increase by *s* ^2^ times. A reconfigurable meta-hologram with up to three picture planes was developed for the meta-hologram (Figure 4c) [84]. The electric field profile modulated by a metasurface will change when stretched by a factor of *s*, which will cause the plane profile to deviate from the former by a factor of *s* ^2^. The holographic pictures in the image plane can change between up to three etchable substrates. Viewing up to three “hidden” images close to the image plane is possible. Since external forces will alter the alignment style and further impact the optical response, LCs-based metasurface can also be manipulated [85, 86]. Rho et al. discovered that two holographic pictures can be shown separately before and after contacting the metasurface in a meta-hologram that responds to surface pressure with a liquid crystal integrated metasurface [87].

These works validate that stretchable substrate-based metasurfaces can serve as an effective and adaptable platform for a range of optical devices that can be reconfigured.

### 3.4 Chemical reaction

The refractive index of the meta-atoms can be purposefully changed along with a substantial change by managing chemical reactions, which updates the optical wavefront [88–91]. Liu et al. engineered chemical processes on the metasurface to produce dynamic meta-holograms at optical frequencies [92]. Magnesium (Mg) nanorods are the main component of the metasurface. Due to Mg’s unique hydrogenation/dehydrogenation properties, the optical characteristic could be changed by manipulating the chemical process that caused the holographic picture to transition. They later improved the chemical reaction technique to produce dynamic OAM holograms [93]. They developed a dynamic meta-hologram using the conducting polymer polyaniline (PANI). Depending on the voltage supplied, PANI would experience considerable changes in its refractive index, transforming the emeraldine and leucoemeraldine states (Figure 4d) [94]. The PANI approach is an excellent method for security applications due to the complex configuration of the chemical reaction process where prompt response is not required.

The transfer of the active characteristics of low-cost, large-scale produced conductive polymers onto optical metasurfaces is an exquisite approach, allowing for the graceful integration of intriguing optical functionalities and material properties, creating novel nano-photonic devices [94].

### 3.5 Separable shares

Separable shares mean splitting the phase information of the hologram into two parts and store them on two carriers, respectively, such as combining a metasurface with a spatial light modulator (SLM) or operating multilayer metasurfaces [95, 96]. By sending the data to the SLM and metasurface, Qu et al. demonstrated the reprogrammable meta-hologram (Figure 4e) [95]. The beautiful design offers two benefits: high information encryption capability and infinitely encoded holographic images. The SLM allows the system tuning to display any holographic image, even when the metasurface is static, greatly enhancing the information capacity. The metasurface is also essential for retrieving the data kept in the system. The data cannot be decrypted without the metasurface, even if someone gets the information encoded in the SLM, protecting the holographic system’s security. Another method that combines multilayer metasurfaces to transport the information was demonstrated by Zentgraf et al. [96]. Each metasurface can function independently in its architecture to recover a holographic image. Different photos will be shown when they are combined with a precise alignment that has been predetermined. Another cryptographic method is offered by translational alignment.

### 3.6 Environment change of meta-holograms

One imitation technique to create a dynamic meta-hologram is to alter the refractive index of the environment [97–100]. A holographic mimicking device based on the phase matrix transformation technique was shown by Liu et al. [97]. The holographic image of such a target will be a “bird” when the sample is in the air. As a comparison, when the environment is adjusted to be oil, it will instantly change to a “fish”. They increased the degrees of freedom and the information storage capability by expanding the system to many wavelengths. Such a design is polarization-sensitive, however. The first polarization-insensitive switchable meta-hologram was then demonstrated by López et al. [98]. The removal of the polarizers from the optical input and output contributes to a reduction in system complexity. In addition to storing information and displaying images, environment-switchable meta-holograms are effective sensors. To create a metasurface-based gas sensor, Rho et al. used the dynamic meta-hologram concept (Figure 4f) [100]. When there is a gas leak, the orientation of the gas-responsive LCs will change instantly, and the optical response of the meta-device will adjust accordingly.

### 3.7 Tunable meta-holograms based on other tuning methods

There are also many other tuning methods to enable the dynamic meta-hologram [101], such as optical pumping [102, 103], micro-electromechanical systems [104], etc. Zhou et al. experimentally demonstrated the dynamic manipulation of THz waves by utilizing a dielectric metasurface [103]. They successfully achieved both mode-selective and mode-unselective control by exciting the system with varying optical wavelengths. This study underscores the promising prospect of employing the wavelength of the incident pump light as an innovative external parameter for dynamically controlling THz waves. This breakthrough can serve as a catalyst for advancing a broad spectrum of tunable meta-devices, each offering a multitude of diverse functionalities.

## 4 Externally programmable meta-holograms

Coding the incident light is being actively investigated to broaden the use of metasurfaces and directly adjust the internal attributes of the metasurface [105–107]. Distinct degrees of freedom (DOFs) that light possesses may be employed as different information routes or as the key to decoding certain information that the light has been encoded with. Modifying the incident beam will significantly expand the capacity for information storage and enhance flexibility. Examples include encoding various holographic pictures into light with orthogonal polarization states with diverse wavelengths. The number of channels will determine how many photos can be stored. The storage capacity will also significantly increase when using additional degrees of freedom in a single meta-hologram. Additionally, it is practical to choose or swap the image to be shown from various channels, opening the possibility of holographic videos [108]. Researching the externally programmable meta-hologram is therefore crucial as well. The following summarizes the manipulations of wavelength, polarization, orbital angular momentum, incidence angle, spatial frequency, and mixtures.

### 4.1 Externally programmable meta-holograms with Single-DOF

**Figure 5:**
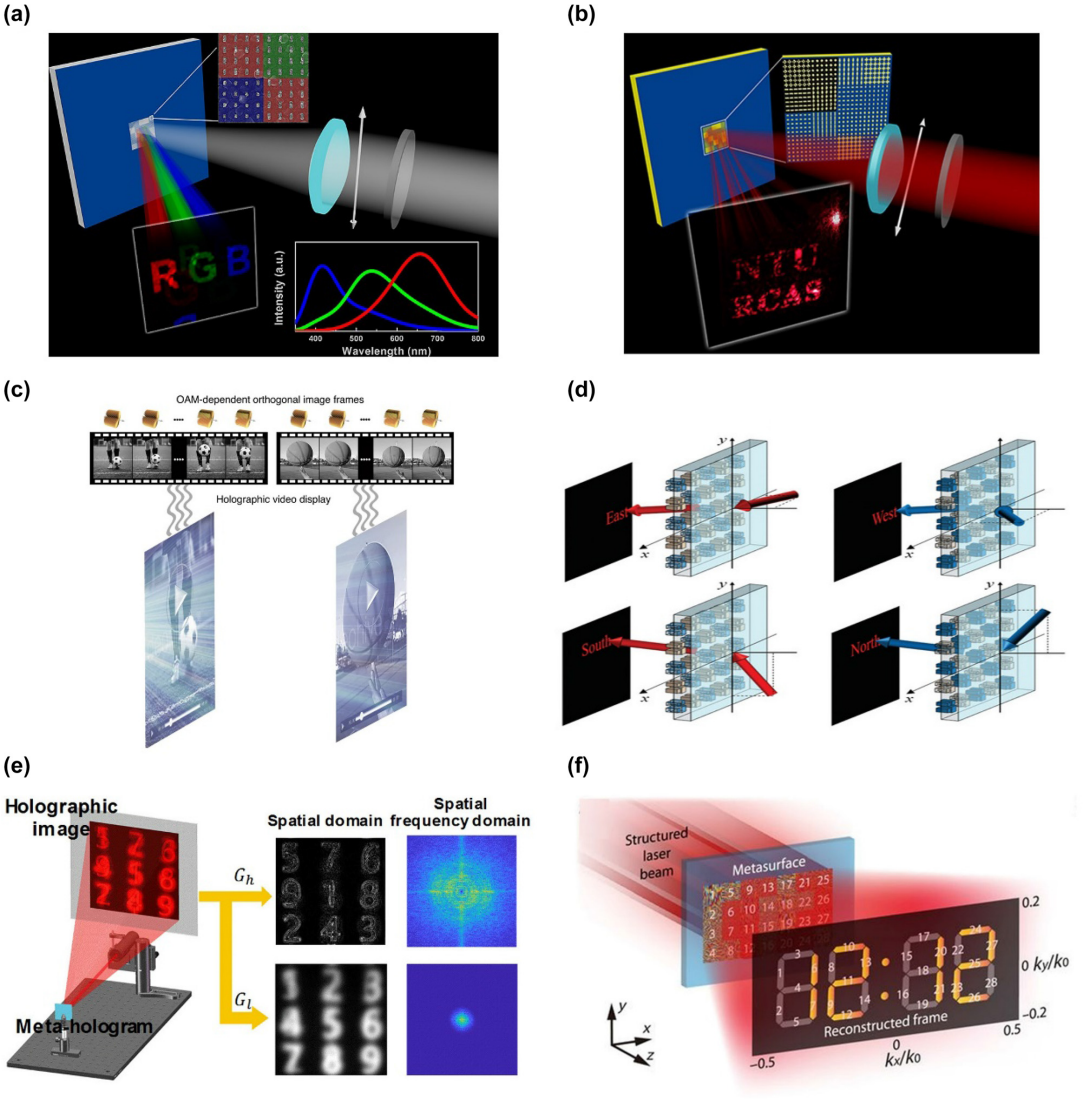
Examples of externally programmable meta-hologram with single DOF. (a) Schematic of the wavelength-controlled meta-hologram. (b) Illustration of the polarization-controlled meta-hologram. (c) Principle of the OAM-controlled meta-hologram. (d) Design of the incident angle-controlled meta-hologram. (e) Schematic of the spatial frequencies-controlled meta-hologram. (f) Principle of the space channel meta-hologram. (a) Reproduced with permission [114]. Copyright 2015, American Chemical Society. (b) Reproduced with permission [124]. Copyright 2014, American Chemical Society. (c) Reproduced with permission [108]. Copyright 2020, Nature Publishing Group. (d) Reproduced with permission [133]. Copyright 2021, Wiley-VCH. (e) Reproduced with permission [135]. Copyright 2019, American Chemical Society. (f) Reproduced with permission [136]. Copyright 2020, American Association for the Advancement of Science.

**Wavelength**: Reconstructing full-color photos using wavelength as a design DOF increases storage capacity [109–113]. A meta-hologram with three hues, for instance, was presented by Tsai et al. in Figure 5a [114]. The letter “*R*” is coded with red paint, the letter “*G*” with green paint, and the color “*B*” with blue paint in the design. The metasurface operates in the reflective mode with an aluminum nanorod as a nano-antenna. Li and colleagues realized that a transmission-mode meta-hologram where three colors were encoded [111]. As a meta-molecule, three-dimensional silicon nanoblocks with various wavelengths are assembled. Similar functions in microwave bands were proposed by Qu et al. utilizing elliptical split resonance rings with different geometrical parameters [115]. At 12, 14, and 16 GHz, three phase-amplitude holographic pictures are created and experimentally confirmed. Additionally, polarization-controlled meta-holograms can increase flexibility and data storage capacity [116–123].

**Polarization**: Another important design DOF to increase the storage capacity is polarization. Tsai et al. demonstrated a reflective-type meta-hologram at visible wavelengths that allowed the holographic picture to be altered between two orthogonal linear polarizations (Figure 5b) [124]. The metasurface’s pixels are comprised of 66 gold nano-antennas of various sizes. The pixel arrays for the two encoded images are also rotated 90° to match the orthogonal polarizations. Circular polarization is also demonstrated. Zhang et al. presented a dynamic meta-hologram at the terahertz band with circular polarization waves instead of linear polarizations [125]. A multi-polarization transmission-type meta-hologram was created by Zentgraf et al. [126]. Up to six holographic pictures might be chosen in a single metasurface using various input/output polarization combinations. A spin-encoded holographic metasurface with dielectric nanoarc structures as its building blocks was proposed by Yuan et al. [127]. The bandwidth and high efficiency of continuous nanoarc structures are superior to their discretized equivalents. Li et al. showed a vectorial holography metasurface made of orthogonal nano-antennas instead of discrete polarization states [128].

**OAM**: OAM is another crucial DOF that provides theoretically infinite multiplexing possibilities [129]. The OAM and the helical wavefront of a propagating beam are related, much as the spin angular momentum and circular polarization are. The OAM-enabled meta-hologram is a promising method to recover an infinite number of holographic images due to the infinite number of orthogonal OAM states. Genevet et al. demonstrated holograms with OAM-multiplexing [130]. With four OAM states ranging from −2 to 2, up to four images can be encoded. However, the foundation of the mentioned research is phase-only SLM, which is susceptible to channel crosstalk. By creating a complex-amplitude metasurface in momentum space, Maier et al. demonstrated innovative OAM-based holography (Figure 5c) [108]. The metasurface might realize up to 200 separate OAM channels in the visible and be fully autonomous in amplitude and phase manipulation. OAM-encoded holograms were made in the microwave domain by Cui et al. [131]. It will contribute to the improvement of imaging and secure communication systems. To further enhance security, Zentgraf et al. induced the polarization channels [132]. Along with the proper OAM mode, the input and output polarization must coincide with displaying the desired holographic image.

**Incident angle**: Meta-holograms also benefit from the light’s incident angle [132–134]. The incidence angle-encoded meta-hologram metasurface was suggested by Lee et al. (Figure 5d) [133]. A single metasurface could recreate four pictures from different incident angles. A spatial frequency-encoded meta-hologram was proposed by Zhang et al. (Figure 5e) [135]. The idea is that various images could be independently coded using high and low spatial frequencies. It is possible to display the desired image using the appropriate digital Gaussian filters. The space channel-enabled meta-hologram may recreate holographic videos with the digital micro-mirror gadget. A dynamic spatial channel multiplexing meta-hologram was proposed by Hong et al. (Figure 5f) [136]. The dynamic meta-hologram may be produced at an incredibly high frame rate (9523 frames per second) in the visible region by properly coding the digital micro-mirror device according to different parts of the metasurface.

Multiplexing meta-holograms leverage a range of degrees of freedom, including wavelengths, polarizations, OAMs, and incident angles, to enhance display efficiency and reduce costs [137]. Reconstructing multiple holographic images using a single metasurface holds excellent potential for various applications and represents an up-and-coming holographic display technology. In the next section, we will delve deeper into the benefits of combining multiple degrees of freedom, which can lead to significant improvements in holographic images and functionality.

### 4.2 Externally programmable meta-holograms with Multi-DOFs

**Figure 6:**
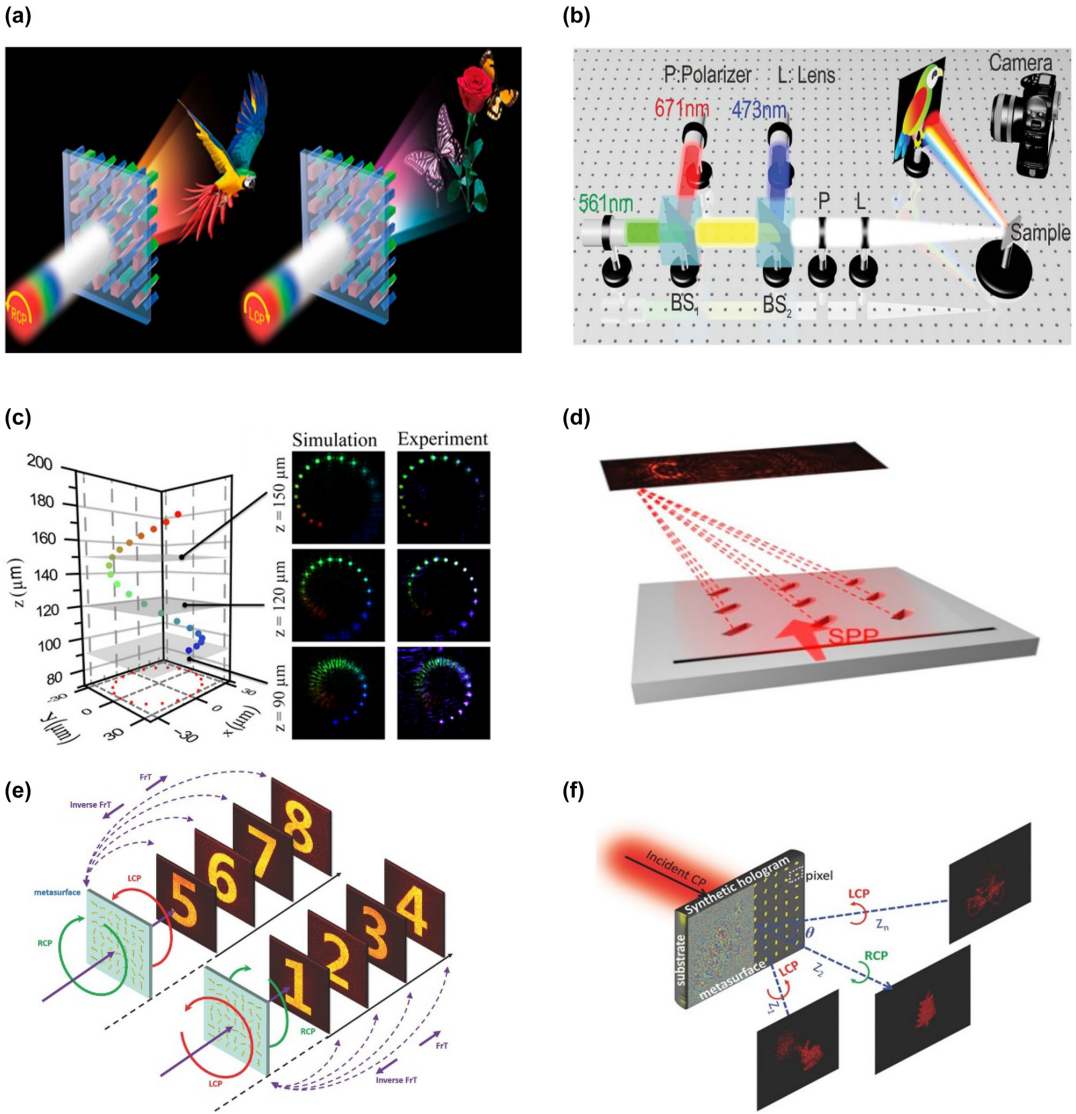
Examples of externally programmable meta-hologram with multi-DOFs. (a) Illustration of the polarization-controlled color hologram. (b) Schematic of the full-color complex-amplitude vectorial meta-hologram. (c) Principle of the off-axis meta-hologram. The insert is the reconstructed holographic image. (d) Schematic (left panel) and the experiment results (right panel) of the meta-hologram. (e) Design of the plasmonic metasurfaces. (f) Schematic illustration of the hybrid multiplexing with a plasmonic metasurface. (a) Reproduced with permission [143]. Copyright 2017, Optica Publishing Group. (b) Reproduced with permission [139]. Copyright 2020, Wiley-VCH. (c) Reproduced with permission [146]. Copyright 2016, American Association for the Advancement of Science. (d) Reproduced with permission [147]. Copyright 2017, American Chemical Society. (e) Reproduced with permission [148]. Copyright 2017, Wiley-VCH. (f) Reproduced with permission [149]. Copyright 2015, Wiley-VCH.

The creation of multi-dimensional manipulation considerably improves the performance of the meta-hologram and stimulates advanced applications, even though the single-dimensional manipulation of optical wavefronts significantly increases its capability. To create a high-dimensional meta-hologram, the two most used coding channels, polarization, and wavelength, are combined [138–142]. A nonlinear metasurface with PB phase support was proposed by Zentgraf et al. [50]. Split ring resonators (SRRs) constructed of gold serve as unit cells. The SRR structure might produce a second harmonic wave because central symmetry is absent. Different images could be reconstructed using extra spins of the fundamental and harmonic beams and polarization combinations by creating the orientation angles of each SRR structure. Such a design broadens optical encryption and anticounterfeiting strategies. Spin-controlled color holograms were suggested by Li et al. (Figure 6a) [143]. The polarization of the incident beam might be changed to alter the full-color holographic images. Such a design offers a way to adjust the holographic image’s color and enhance optical storage capacity. Chu et al. created a metasurface combining left/right circular polarizations and three wavelengths to encode holographic images into distinct channels [144]. Information is encoded into other channels to increase security. Polarization-controlled trichromatic holographic pictures were produced by Duan et al. [145]. Different incident polarizations could result in a variety of picture hues. A vectorial full-color hologram image can be produced by prearranging several wavelengths as the desired polarization, which improves data storage, encryption, and anti-counterfeiting even more. Li et al. showed that a simple single-sized meta-atom metasurface independently controls light’s phase, amplitude, and polarization. The guiding design idea produced full-color, polarization-controlled holographic images (Figure 6b) [139]. Various full-color images can be produced by changing the input/output polarization combinations.

In addition, there are numerous additional crucial combinations, such as wavelengths and light’s incident angle. An incidence angle-controlled multi-color meta-hologram was proposed by Luo et al. (Figure 6c) [146]. Various holographic images would be displayed by altering the incident angle and wavelength. They also show a multi-color 3D meta-hologram in their work. Surface plasmon polariton (SPP) propagation and polarization scattering were used by Zhu et al. to suggest a meta-hologram (Figure 6d) [147]. The SPP wave will propagate in various directions because of the placement of multiple polarizers at the incident and image planes, and corresponding holographic images can be produced. A position and polarization-encoded meta-hologram, which causes several pictures to emerge in various image planes, was proposed by Wang et al. (Figure 6e) [148]. The polarization, location, and incidence angle-encoded meta-hologram by Zentgraf et al. considerably increased the DOFs for the programmable meta-hologram (Figure 6f) [149]. The multiplexing meta-hologram can achieve high-quality, nondestructive readout, and significantly increase the information capacity by incorporating different DOFs into a single meta-device.

## 5 Conclusions and perspectives

In conclusion, we have discussed the most recent developments in intrinsically and externally programmable meta-holograms. The internal programmable meta-hologram can achieve dynamic control over the optical responses under external excitations compared to the static meta-hologram. The external meta-hologram can encode many images into a single metasurface. Table 1 shows the summary of various meta-holograms regarding features and challenges. The metasurface is superior to conventional holograms in several ways, including sub-wavelength resolution, a wide field of view, and ultra-compactness. The review of typical scientific works on programmable meta-holograms based on two mechanisms—imposing external incentives on the metasurfaces and multiplexed metasurfaces with multiple DOFs made possible by integrated resonant units—was conducted in the literature [150, 151]. From the description above, programmable meta-holograms significantly increase the variety of hologram applications, including optical encryption, information storage, and image/video projection, and enable the metaverse.

**Table 1:** Summary of various meta-holograms regarding features and challenges.

Methods	Outstanding features	Challenges
Electricity	Large-scale, low-cost, and high-performance	Not applicable in the visible
Thermal	High security	Limited images
Strain	Modulating hologram image plane	Limited images, low switch speed
Chemical reaction	High security	Little images, low switch speed
Separable shares	Unlimited images	Need extra bulky elements
Changing environment	Mimicry behavior	Limited images, low switch speed
Single-DOF	Multi-images in single metasurface	Limited images except for space channel and OAM
Multi-DOFs	Expanded degrees of freedom, high security	

With the continuous development of programmable meta-holograms, our comprehension of basic physics fundamental concepts will help the development of related sciences. Many other metasurface-based applications can benefit from understanding how external stimuli, because of the greater capacity to regulate the optical wavefront, such as electricity and temperature, aid in producing a holographic image. The high-dimensional multiplexing technology will also be applied in several circumstances, including parallel data processing and optical communication. The visible display of holographic video and highly switchable images, both necessary for the metaverse, is still challenging. Researchers have shown some advancements. The reprogrammable meta-hologram dramatically improves optical information encryption and authentication by enabling arbitrary real-time holographic images and films supported by SLM [95]. Due to the theoretically infinite number of orthogonal OAM modes of light, the complex-amplitude OAM meta-hologram enables holography video utilizing a single metasurface [98].

Advancements in meta-hologram technology are paving the way for the development of the metaverse, enabling immersive, lifelike experiences that blur the line between physical and virtual reality. Enhancing image quality and downsizing metaverse equipment can significantly improve user experience. There is still much space for development. The three-dimensional color holographic display effect with programmable multi-channel switching has been achieved using sophisticated amplitude modulation and information multiplexing techniques. However, the actual use of metasurface holography is still constrained by issues like low refresh and frame rate and complex optical pathways. Another significant issue is the fabrication component. Traditional processing methods like EBL, FIB, and others have drawbacks, including high costs and processing times. These do not allow for the widespread commercial use of meta-devices in the metaverse.

We anticipate several promising avenues in developing and implementing programmable meta-holograms, with the potential to significantly impact the metaverse’s advancement. One critical step in this endeavor is the fusion of metasurface physics with quantum technologies [152]. Quantum devices can be fundamental building blocks for achieving secure quantum communication and information systems, thereby providing heightened security for meta-holograms in the metaverse. Furthermore, leveraging quantum technology has the potential to enhance the display resolution of meta-holograms [153]. The application of artificial intelligence in the design process of programmable meta-holograms can simplify the design complexity and facilitate the attainment of high-dimensional functionalities. Integrating intrinsic and external control mechanisms holds promise as a viable approach for advancing programmable optical meta-holograms. For instance, Cui et al. exemplified a methodology wherein, through the encoding of distinct sequences, the metasurface exhibits the capacity to reconstruct arbitrary holographic images across two polarization channels [154]. This observation underscores the ability to represent different polarization states through specific sequences, leading to the emergence of distinctive characteristics within the resulting reconstructed holographic images.

The metaverse systems can use the real-time dynamic three-dimensional full-color meta-hologram display scheme to add new capabilities and create a tiny configuration. The programmable optical meta-hologram offers exceptional tunability, enabling it to display user-selected content and reproduce various scenarios. This presents both an opportunity and a challenge. While theoretically possible, several practical hurdles exist to overcome for successful commercialization. Currently, most tunability solutions excel in wavelength ranges beyond the visible light spectrum, hampered by manufacturing constraints. Creating a programmable optical meta-hologram with high efficiency, high modulation depth, and speed is also a challenging task, and it typically involves advanced research and engineering in photonics, nanotechnology, and materials science. Achieving these goals requires a combination of innovative design, specialized materials, and precise control mechanisms. Achieving high efficiency and modulation depth requires careful design and simulation during metasurface optimization. High-speed modulation can be accomplished using various techniques, such as optical pumping [103] or MEMS-based metasurfaces [104]. Once these issues are resolved, the metaverse’s extensive commercial applications will benefit from the scientific progresses. As a result, more people will benefit from cutting-edge technology. The adjustable and dynamic holographic frames provided by the ultra-flat meta-device will significantly improve the user experience.
